# Exploring the lived experience of loneliness and social isolation in informal palliative caregivers: A systematic review

**DOI:** 10.1177/26323524251397000

**Published:** 2025-12-04

**Authors:** Louisa Cole, Tracy Collins, Renée Speyer, Cara Ellis, Reinie Cordier

**Affiliations:** 1 Northumbria University, Newcastle upon Tyne, UK; 2 University of Galway, Ireland

**Keywords:** loneliness, social isolation, palliative care, end-of-life care, informal caregivers, qualitative research, systematic review

## Abstract

**Introduction:**

Loneliness and social isolation negatively affect an individual’s mental and physical health. Although there is literature exploring loneliness in informal caregivers and literature exploring the unique challenges of providing informal palliative care, there is no existing literature with an explicit focus on loneliness and social isolation in informal palliative caregivers. This systematic review aims to explore the experiences of loneliness and social isolation in informal palliative caregivers.

**Methods:**

The databases of CINAHL, Embase, PsycINFO and PubMed were searched on 23 October 2024. Qualitative literature that studied loneliness and/or social isolation in adult informal palliative and/or end-of-life caregivers was included. Quantitative and non-English language literature was excluded. The studies were screened, and the literature was analysed using thematic analysis. The included studies were critically appraised using the CASP Qualitative Studies Checklist.

**Results:**

Of all the articles screened, 28 were included in the review. The total number of participants across the studies was 505. Three overarching themes (with subthemes) were identified from the analysis. The themes were Caring is complex (19 studies), Lack of support (14 studies) and What helps (9 studies).

**Discussion:**

The challenging and unique experiences of providing palliative care can lead to or exacerbate feelings of loneliness and social isolation. Caregivers struggle with managing the demands of palliative care while having little support during the patient’s illness and after they have died. Social support and faith practices alleviated feelings of loneliness for some caregivers. The critical appraisal identified issues around ethical considerations and the researcher–participant relationship. Practitioners should support caregivers to develop meaningful occupations that allow for social connection.

## Introduction

Loneliness is a negative and painful emotional state due to a perceived lack of quality or quantity of social relationships.^1,2^ It is often associated with feelings of unhappiness, restlessness and despondency.^3^ Loneliness and social isolation are related to similar concepts, such as social networks and alienation; however, they are distinct.^2^ Social isolation is often defined as an objective lack of social contact,^1^ with the limited social interactions not being meaningful or fulfilling.^4^ Being alone does not necessarily result in loneliness, and loneliness can be felt in the presence of other people.^5^ However, there are varying definitions of the concepts across the literature. Although social isolation is frequently defined as the objective counterpart to loneliness, some literature interprets the concept more subjectively. For example, Hajek et al.^6^ define social isolation as the feeling of not belonging to society, rather than an objective lack of social connections. Thus, suggesting further overlap between the concepts.

Loneliness and social isolation can have a negative effect on an individual’s health. A study investigating the association between loneliness and social isolation on health outcomes in older adults surveyed approximately 7000 participants.^7^ The study found that those who were lonely and socially isolated were the least healthy, had the highest rates of hospitalisation and the lowest quality of life scores.^7^ In a review and meta-analysis, loneliness was found to have a medium to large effect on all health outcomes.^8^ It has the largest impact on mental health and well-being.^8^ However, these results should be interpreted with caution, as they only show a correlation. There is no established causal relationship between loneliness and health outcomes.^8^ The relationship is complex, with several factors influencing each other.^8^

Loneliness is significantly associated with providing informal care, as found in a systematic review conducted by Hajek et al.^6^ Palliative caregivers experience loneliness. For example, Dahlborg Lyckhage and Lindahl^9^ interviewed six palliative caregivers and found that their lives were defined by loneliness as they felt abandoned with too much responsibility, with similar findings reported by Ateş et al.^10^ In a mixed methods study of 87 palliative family caregivers, most of the participants reported that their caregiving responsibilities left them socially isolated and emotionally distressed.^10^

Palliative care is a patient-centred approach to care for people with an advanced or terminal illness and their loved ones.^11^ A terminal illness is a progressive disease that does not respond to treatment; a cure is no longer possible.^12^ Palliative care teams involve many healthcare professionals who work alongside the informal caregiver.^13^ Palliative care aims to optimise quality of life, including pain management and psychological support.^14^ End-of-life care is part of palliative care that occurs when there is rapid deterioration and increased symptoms, generally when life expectancy is less than 12 months.^12^ The focus of end-of-life care is to allow the patient to die with dignity.^14^ In this review, the term ‘palliative caregivers’ will describe participants in all the included studies, as palliative care encompasses end-of-life care. For clarity, the term ‘patients’ will be used to describe the loved ones who are being cared for.

Every country is experiencing an increase in the proportion and number of older people in their population.^15^ As populations age, the number of people with life-limiting illnesses increases.^16^ This highlights the importance of having skilled people who can provide care. The need for palliative care is estimated to increase by 87% by 2060, as reported by the World Hospice Palliative Care Alliance.^17^ Thus, the health and well-being of informal palliative caregivers are essential on a societal and individual level.

Stajduhar^16^ reports that informal caregivers experience greater care demands during the end-of-life phase of the illness; with the toll of these demands extending into caregiving. This could be due to the unique challenges and responsibilities that come with palliative care. A systematic literature review exploring the role of informal caregivers in palliative care was conducted by Reigada et al.^18^ The study found seven major palliative caregiving roles, including the activities and tasks that make up those roles.^18^ Although there was some overlap with non-palliative caregivers, there were responsibilities specific to palliative and end-of-life care. For instance, they must face their own mortality while supporting the patient, make critical end-of-life decisions and assist in the dying process in hopes of providing a peaceful death.^18^ Therefore, it is important to understand and support this specific population that faces unique and increasing challenges.

This concern has been echoed within the scientific community, which identified understanding the needs of informal caregivers as a palliative care research priority.^19^ This was due to the holistic approach that palliative care takes and the support system that the caregivers provide to the patient.^19^ In their narrative review of the loneliness of cancer caregivers, Gray et al.^20^ suggested that further research is needed to identify the factors that protect against or increase loneliness in informal caregivers. The aims of this study are shaped by the lack of contemporary literature reviewing the experiences of loneliness and social isolation in informal palliative caregivers. As previously discussed, due to the unclear distinctions and varying definitions across the literature, the current study will explore both concepts to broaden the understanding of the phenomena. Therefore, the current study aims to explore the experience of loneliness and social isolation within the occupational role of being an informal palliative caregiver. The first objective is understanding how informal palliative caregivers experience loneliness and social isolation. The second objective is to identify practices that help to alleviate loneliness and social isolation in palliative caregivers.

## Methodology

The current systematic review has been reported according to the Preferred Reporting Items for Systematic Reviews and Meta-Analyses (PRISMA) guidelines.^21^ The completed checklists for writing an abstract (Supplemental Table 1) and reporting a systematic review (Supplemental Table 2) can be found in the Supplemental Material.

### Eligibility criteria

The studies had to be original qualitative or mixed methods with relevant qualitative findings to be eligible for inclusion. The included studies had to investigate the perspectives and experiences of adults (⩾18 years old) who are informal caregivers providing palliative or end-of-life care. The study could be from any country, as loneliness and social isolation are experienced globally. The study needed to explore loneliness and/or social isolation related to experiences of palliative or end-of-life caregiving.

Literature not published in English was excluded, as this review did not have the resources to translate papers. Studies related to professional palliative caregivers were excluded. Quantitative literature was excluded as this methodology does not collect the in-depth data required to answer the research question. Articles with no original research findings, such as commentaries, were also excluded.

### Information sources and search strategies

A search was conducted to identify relevant literature. The search terms were applied CINAHL, Embase, PsycINFO and PubMed databases. Subject headings (e.g. MeSH or Thesaurus terms) related to three concepts (i.e. loneliness and social isolation, palliative care and caregivers) were combined to retrieve relevant search results. See Table 1 for key search strategies. Each database was last searched on 23 October 2024.

**Table 1.** Key search terms across all databases.

Search strategies	Number of records
*CINAHL Ultimate (EBSCO)*: ((MH ‘Loneliness’) OR (MH ‘Loneliness (Iowa NOC)’) OR (MH ‘Social Alienation’) OR (MH ‘Social Isolation’) OR (MH ‘Social Isolation (NANDA)’) OR (MH ‘Social Isolation (Saba CCC)’) OR (MH ‘Bereavement’) OR (MH ‘Caregiver Burden’)) AND ((MH ‘Palliative Care’) OR (MH ‘Palliative Care Nursing’) OR (MH ‘Palliative Medicine’) OR (MH ‘Palliative Care Nurses’) OR (MH ‘Hospice (Saba CCC)’) OR (MH ‘Hospice Care’) OR (MH ‘Hospice Nursing’) OR (MH ‘Hospice Patients’) OR (MH ‘Hospice Nurses’) OR (MH ‘Terminally Ill Patients’)) AND (MH ‘Caregivers’) Limit: English	564
*Embase (Ovid)*: (loneliness/ OR alienation/ OR social isolation/ OR bereavement/ OR caregiver burden/) AND (palliative nursing/ OR palliative therapy/ OR terminal care/ OR terminal disease/ OR hospice/ OR hospice care/ OR hospice nursing/ OR hospice patient/ OR terminally ill patient/ OR incurable disease/) AND Caregiver/ Limit: English language	1087
*PsycINFO (Ovid)*: (loneliness/ OR alienation/ OR social isolation/ OR Bereavement/ OR caregiver burden/) AND (palliative care/ OR terminally ill patients/ OR hospice/) AND (Caregivers/ OR Caregiving/) Limit: English language	343
*PubMed*: (‘Loneliness’[Mesh] OR ‘Social Alienation’[Mesh] OR ‘Social Isolation’[Mesh] OR ‘Caregiver Burden’[Mesh] OR ‘Bereavement’[Mesh]) AND (‘Hospice and Palliative Care Nursing’[Mesh] OR ‘Palliative Care’[Mesh] OR ‘Hospice Care’[Mesh] OR ‘Terminal Care’[Mesh] OR ‘Hospices’[Mesh] OR ‘Terminally Ill’[Mesh]) AND (‘Caregivers’[Mesh]) Limit: English	515

### Selection process

**Figure 1.**
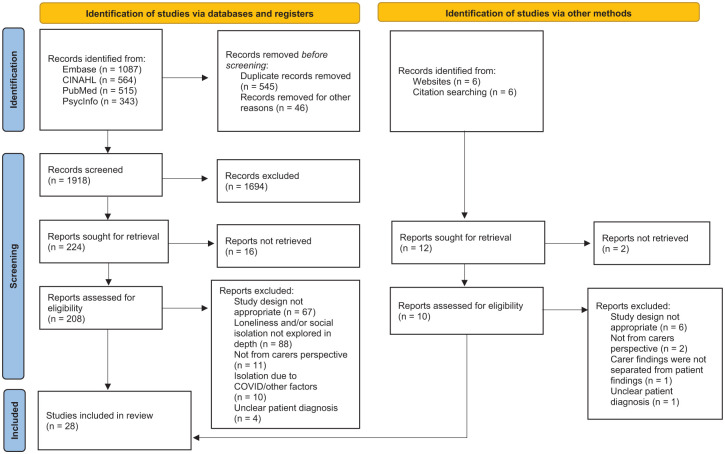
PRISMA flowchart showing the selection of studies for review. PRISMA, Preferred Reporting Items for Systematic Reviews and Meta-Analyses.

All the identified records were imported to EndNote software.^22^ Duplicates were removed. The titles and abstracts of the studies were screened independently by two reviewers. The reviewers discussed and resolved any conflicts regarding the inclusion and exclusion of individual studies. The full-text versions of the eligible studies were retrieved. These studies were assessed against the inclusion and exclusion criteria by both reviewers. The included studies were referenced checked for other relevant studies. Websites that were relevant to the three concepts (i.e. loneliness and social isolation, palliative care and caregivers) were searched. The study selection process is shown in Figure 1.

### Data collection process

The data across all the included studies were extracted using comprehensive data extraction forms. Data included country of origin, study design, sample size, participant age, participant gender, participant employment status, patient diagnosis and the phenomena of interest (i.e. loneliness and social isolation, palliative care and caregivers). Illustrative quotations were extracted from the results of the studies. A review protocol was not prepared. All data extracted from included studies and used in the review are contained in the review.

### Study risk of bias assessment

All the studies meeting the inclusion criteria had their methodological quality assessed using the CASP Qualitative Studies Checklist.^23^ The checklist specifically assesses the study’s results, their validity and local value. The tool was chosen due to its suitability for use in qualitative health-related research, with endorsements from the WHO and Cochrane.^24^

### Qualitative synthesis methods

The studies’ findings, including themes, subthemes and illustrative quotations, were synthesised using thematic analysis.^25^ Some findings were excluded from synthesis due to a lack of relevance to the aims of the current research study. Inclusion criteria for the findings were explicit discussions of loneliness and social isolation or other related concepts such as aloneness. Exclusion criteria included findings that discussed patient experiences or caregiver burdens unrelated to loneliness and social isolation, such as physical health issues.

The first author read the included studies multiple times to become familiar with the data. Initial codes were generated based on features that appeared across the data set. Potential themes were created by collating similar codes. The potential themes were reviewed and discussed with the second author to ensure they were clearly defined and relevant to the data set. This process was conducted based on Braun and Clarke’s^25^ thematic analysis. This was chosen due to its flexibility and well-structured steps (King,^26^ p. 257). Thematic analysis can provide a detailed and complex account of the data, which is valuable when exploring a nuanced experience.^25^

## Results

### Study selection

Of the 1918 records screened, 208 full-text articles were assessed using the inclusion and exclusion criteria. A total of 28 studies met the inclusion criteria and were included in this review. See the PRISMA flowchart (Figure 1) for full details.

### Study characteristics

All the studies included in this systematic review collected qualitative data. The two approaches used by the studies were phenomenology and grounded theory. All the studies interviewed their participants. Two studies used focus groups alongside interviews.^27,28^ One study collected data through diary entries and interviews.^29^ One study collected data solely through diary entries.^30^ The total sample size of the combined studies was 505 participants. Not all the studies reported their participants’ ages. The youngest reported age was 18, and the eldest was 87. There were 320 female and 141 male participants across the studies. There were 44 participants who did not have their gender recorded. Not all the studies reported the employment status of their participants. However, of those who did, 37% were employed compared to 63% who were unemployed. The studies were conducted in a range of countries, including the United States (*n* = 5), the Netherlands (*n* = 4), Canada (*n* = 4), Sweden (*n* = 4), Australia (*n* = 4), United Kingdom (*n* = 2), Northern Ireland (*n* = 1), Malaysia (*n* = 1), Republic of Ireland (*n* = 1), Croatia (*n* = 1), Norway (*n* = 1), Saudi Arabia (*n* = 1) and Brazil (*n* = 1). One study was conducted in South Africa and Uganda.^31^ The three most common diagnoses for the person receiving care were cancer, Parkinson’s and chronic obstructive pulmonary disease. See Table 2 for full details.

**Table 2.** The characteristics and demographics of the included studies.

Author (date)	Country	Risk score	Study design	Sample size	Caregiver age	Caregiver gender			Employment status	Patient diagnosis
						Female	Male	Not reported		
Baumgardner and Mayo (2021)	USA	Low	Qualitative, phenomenology, interviews	10	M: 67.1 R: 45–84	8	2		Employed: 1, unemployed: 9	Dementia
Beng et al. (2013)	Malaysia	Medium	Qualitative, interviews	15	NR	10	5		Employed: 5, unemployed: 10	Cancer, non-cancer illnesses
Benites et al. (2023)	Brazil	Low	Qualitative, longitudinal, phenomenology, interviews	10	M: NR R: 22–77	7	3		NR	Cancer
Bilić et al. (2022)	Croatia	Low	Qualitative, phenomenology, interviews	8	M: 63.7 R: 38–75	7	1		NR	Cancer, dementia, prostate adenoma, COPD
Bouchal et al. (2015)	Canada	Low	Qualitative, hermeneutic phenomenology, interviews	8	M: NR R: 55–81	2	6		NR	Cancer
Broom et al. (2019)	Australia	Low	Qualitative, longitudinal, interviews	15	M: NR R: 40–80	10	5		NR	Cancer
Cain et al. (2004)	Canada	Low	Qualitative, focus groups, individual interviews	42	NR			42	NR	Cancer, HIV-related illnesses
Collins et al. (2016)	Australia	Low	Qualitative, interpretive phenomenology, interviews	14	M: NR R: 25–51	12	2		Employed: 4, unemployed: 10	Leukaemia, cystic fibrosis, Sandhoff disease, xanthoastrocytoma, central congenital hypoventilation syndrome, multicystic bilateral renal dysplasia, Schwartz-Jampel syndrome, degenerative neurological condition, nemaline rod myopathy, Lennox–Gastaut syndrome, severe cerebral palsy and serious undiagnosed neurological disability
Fenton et al. (2023)	USA	Low	Qualitative, interviews	19	M: 64 R: 40–82	14	5		NR	Cancer
Fitzsimons et al. (2019)	UK	Low	Qualitative, interviews	30	NR	15	15		NR	Heart failure
Fox et al. (2017)	Republic of Ireland	Low	Qualitative, interviews	12	M: 68.2 R: 58–78	11	1		NR	Parkinson’s
Gibson et al. (2019)	Canada	Low	Qualitative, interviews	10	M: 81.6 R: NR	8	2		NR	Dementia
Haan et al. (2021)	Netherlands	Low	Qualitative, grounded theory, interviews	28	M: 58.1 R: 23–84	16	12		NR	Cancer, organ failure
Hafez et al. (2022)	Saudi Arabia	Low	Qualitative, phenomenology, interviews	24	M: 34.3 R: 18–47	24			Employed: 4, unemployed: 20	Congenital anomalies, neurodegenerative disease, cancer
Hasson et al. (2010)	Northern Ireland	Low	Qualitative, exploratory descriptive, interviews	15	NR	4	11		NR	Parkinson’s
Hebdon et al. (2023)	USA	Low	Qualitative, audio diaries	50	M: 57 R: 30–86	38	12		Employed: 23, unemployed: 26, NR: 1	Cancer
Heidenreich et al. (2014)	Australia	Low	Qualitative, exploratory descriptive, interviews	5	M: NR R: 50–65	5			Unemployed: 5	NR
Holtslander and Duggleby (2008)	Canada	Low	Qualitative, interpretive, descriptive, interviews, diary entries	13	M: NR R: 60–79	13			NR	Cancer
Keesing S et al. (2011)	Australia	Low	Qualitative, interviews	14	M: 54.9 R: 25–71	13	1		NR	Cancer, cardiac disease, renal disease, COPD, MS
Lennaerts-Kats et al. (2020)	Netherlands	Low	Qualitative, interpretive phenomenology, interviews	10	M: NR R: 44–81	8	2		NR	Parkinson’s
Linderholm and Friedrichsen (2010)	Sweden	Low	Qualitative, hermeneutic phenomenology, interviews	13	M: 58.1 R: 38–78	8	5		Employed: 8, unemployed: 5	Cancer
Mason and Hodgkin (2019)	Australia	Low	Qualitative, phenomenology, interviews	10	M: NR R: 55–87	4	6		Unemployed: 10	Cancer, non-cancer illnesses
Nissmark and Fänge (2020)	Sweden	Low	Qualitative, interviews	6	M: NR R: 22–56	4	2		Employed: 4, unemployed: 2	Cancer
Proot et al. (2003)	Netherlands	Low	Qualitative, grounded theory, interviews	13	M: 51 R: 28–80	11	2		NR	Cancer
Staats et al. (2024)	Norway	Low	Qualitative, descriptive, exploratory, interviews	24	NR	15	9		NR	NR
Strang et al. (2018)	Sweden	Medium	Qualitative, interviews, focus groups	35	NR	10	25		NR	COPD
Streid et al. (2014)	South Africa, Uganda	Low	Qualitative, interviews	37	M : 44.8 R: 19–77	32	3	2	NR	Cancer, HIV
Totman et al. (2015)	UK	Low	Qualitative, interviews	15	M: 50.2 R: 27–66	11	4		Employed: 10, retired or unemployed: 5	Cancer

COPD, chronic obstructive pulmonary disease; HIV, human immunodeficiency virus; M, mean; MS, multiple sclerosis; NR, not reported; R, range.

The studies in this review were selected due to their discussions of loneliness (*n* = 10) and social isolation (*n* = 10). Eight of the studies discussed both loneliness and social isolation. The studies were selected because their participants were caregivers of people with terminal illnesses. The participants in nine of the studies provided palliative care. The participants in 11 of the studies provided end-of-life care. In five of the studies, participants provided palliative and end-of-life care. No distinct patterns between palliative care and end-of-life care experiences were found in the thematic analysis. See Table 3 for full details.

**Table 3.** The content of each study related to loneliness and social isolation and form of care.

Author (date)	Loneliness/social isolation			Form of care	
	Loneliness	Social isolation	Both	Palliative	End of life
Baumgardner and Mayo (2021)		X		X	
Beng et al. (2013)	X			X	
Benites et al. (2023)	X				X
Bilić et al. (2022)	X			X	
Bouchal et al. (2015)	X				X
Broom et al. (2019)		X		X	X
Cain et al. (2004)		X		X	X
Collins et al. (2016)		X		X	
Fenton et al. (2023)		X			X
Fitzsimons et al. (2019)			X	X	X
Fox et al. (2017)			X	X	
Gibson et al. (2019)	X				X
Haan et al. (2021)	X			X	X
Hafez et al. (2022)		X			X
Hasson et al. (2010)			X	X	X
Hebdon et al. (2023)	X				X
Heidenreich et al. (2014)			X	X	
Holtslander and Duggleby (2008)	X				X
Keesing et al. (2011)			X		X
Lennaerts-Kats et al. (2020)			X		X
Linderholm and Friedrichsen (2010)			X		X
Mason and Hodgkin (2019)			X		X
Nissmark and Fänge (2020)		X		X	
Proot et al. (2003)	X				X
Staats et al. (2024)	X				X
Strang et al. (2018)		X		X	
Streid et al. (2014)		X		X	
Totman et al. (2015)		X			X

### Data items

Palliative care was similarly defined across the studies. The consensus was that palliative care is a holistic approach to care that requires a multidisciplinary team.^32–35^ The focus of palliative care is the physical, emotional and spiritual well-being of the patient and their family.^32,36,37^ It is applicable to all stages of a life-limiting illness.^38^ There was minimal distinction between the definitions of palliative care and end-of-life care in the studies. The main difference was the emphasis placed on the comfort of the patient during end-of-life care as the symptoms of the illness advanced.^39,40^

Loneliness and social isolation were defined more distinctly, although there were similarities. The definitions of loneliness focused more on the emotional burden and feelings of distress it induces.^41^ It was described as limited social support with no meaningful connection, leading to feelings of alienation and abandonment.^34,40^ Social isolation was defined with more focus on the tangible aspects of care. The caregiver is often contained in their home, secluded from the wider community, and has significant limitations on their availability to socialise.^33,42^

### Risk of bias in studies

Every study included in this systematic review was appraised using the CASP Qualitative Studies Checklist.^23^ See Supplemental Table 3 for full details. The results of the checklist were used to inform the discussion of the methodology quality of the studies. No studies were removed from the review based on methodological limitations. A risk of bias score for the studies was calculated by assigning a number to each of the three possible answers (yes = 0, unclear = 1, no = 2) to questions 1–9 on the checklist. The studies were identified as either low, medium or high risk, depending on their total score and which range they fell into. Studies scoring 0–6 were considered low risk, those scoring 7–12 were considered medium risk, and high risk was between 13 and 18. The risk score of each study is shown in Table 2. Twenty-six studies were scored as low risk, and two were scored as medium risk.

The quality appraisal identified several limitations of the studies included in the review. Sixteen studies did not report any consideration of the relationship between the participants and the researchers, including a critical examination of their influence on the study. This is important to consider as the participants of the studies are likely to be vulnerable, particularly caregivers who were recently bereaved.^43^ Another limitation was that 15 studies did not clearly report the ethical considerations. Vergnes et al.^44^ argue that if the ethics of included studies are inadequate, it raises concerns about the moral acceptability of using the results in a systematic review. However, whether these limitations are due to genuine methodological issues or absence in reporting is unclear.

### Results of syntheses

**Figure 2.**
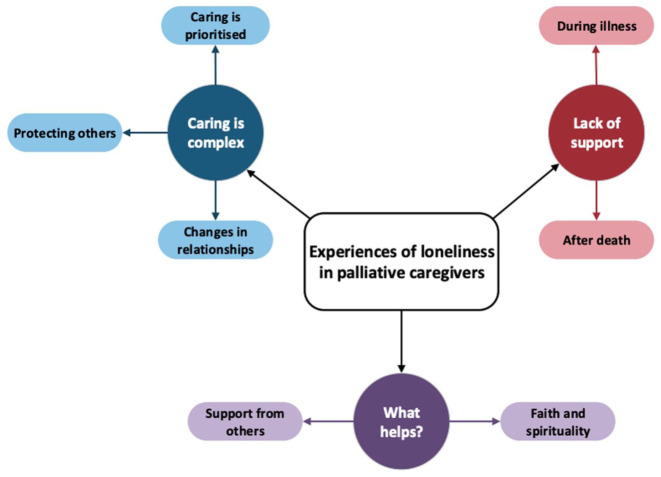
Themes and subthemes identified in the review.

Three main themes were identified. These were ‘Caring is complex’, ‘Lack of support’ and ‘What helps?’ Each overarching theme had associated subthemes. The themes and subthemes are illustrated in Figure 2.

#### Caring is complex

The first theme identified was ‘Caring is complex’. Nineteen of the studies illustrated this theme. This theme incorporates three subthemes, which are ‘Caring is prioritised’, ‘Changes in relationships’ and ‘Protecting others’. The theme illustrates the conflicting and nuanced experiences of providing palliative care to a loved one.

#### Caring is prioritised

This first subtheme is ‘Caring is prioritised’ and is found in 11 of the reviewed studies. It focuses on the caregivers’ overwhelming responsibility to provide care and duty to be available to the patient 24 h a day.^27,31^

I didn’t do anything, I was exhausted all the time . . . there was no energy left to do anything just for me or even see your friends. (Collins et al.,^33^ p. 503)

Caring is time-consuming and often prioritised over other obligations.^28,34,37,42^ Carers give up their work, hobbies and friendships.^33,34,39,45^ Carers must be constantly available to the patient, and they can rarely leave their home.^27,31,33^ Due to the burden of caring, caregivers often did not have the energy or time to invest in other relationships.^28,46^ As the patient deteriorates, their needs increase, which further isolates the caregiver.^27,32^ Thus, caregivers felt lonely and struggled to manage their caregiving responsibilities alone. However, one group of participants purposefully withdrew from social events. They were end-of-life caregivers of children and found socialising insignificant.^39^ For some caregivers, time and energy constraints were not the only barriers to occupations outside of caregiving. Some caregivers fear being perceived as a burden by others and uncomfortable interactions regarding death and illness as reasons why they purposefully isolate themselves.^28,33^

#### Protecting others

The second subtheme is ‘Protecting others’, which was found in seven of the studies. It describes the experience of palliative caregivers in which they keep information and their feelings to themselves to avoid distressing those they love. The subtheme was illustrated in a quotation from a caregiver speaking about her spouse.^41^

We get more upset, sadder, but you can’t show it to him either. I think he gets more upset. (Benites et al.,^41^ p. 254)

Caregivers often felt protective over the patient, especially as their relationships changed. Caregivers wished to protect the patient from further emotional distress so they would not share their feelings with them.^30,35,41,47^ Caregivers did not want to discourage patients about their prognosis or induce feelings of guilt by discussing the burden of caregiving.^30,34,42^ This protection extended to other family members and friends.^30^ Carers kept their difficulties to themselves to not worry the friends and family, as well as out of fear of being a burden.^30,47^ Caregivers were left to cope with their emotions in isolation, which exacerbated their feelings of loneliness.^30,47^ This experience often happens simultaneously with caregivers prioritising the patient by spending all their time and energy providing care, as discussed in the previous subtheme. This reflects the complications of providing palliative care. The caregivers are experiencing loneliness by prioritising care; however, they do not share this burden with others even if it may alleviate their feelings of loneliness.^30,31,47^

#### Changes in relationships

This subtheme depicts the changes to the relationship between the caregiver and the patient, as their original roles and needs alter due to the patient’s illness and care requirements.^38,42^ This theme was found in six of the studies.

Although he is sitting next to me, but I feel like I’ve lost my husband. He’s not talking to people. (Beng et al.,^46^ p. 478)

Caregivers lost the companionship of the patient.^34,36,38,42^ The relationship with the person they were caring for shifted away from their original dyad and towards a primarily caregiver–patient relationship.^42^ This was suggested to be a result of the changes in the patient’s symptoms and capacity.^36,38,40,48^ This was especially pertinent for caregivers of people with dementia and Parkinson’s when the patients lost their ability to communicate verbally.^40^ This difficulty in maintaining a relationship led to feelings of loneliness. As discussed earlier, carers prioritise caregiving over other obligations; thus, they spend most of their time with the patient. This resulted in the loss of other friendships and social contacts, which created further feelings of social isolation during the bereavement period; carers had very few people to share their feelings with.^36^

#### Lack of support

The second theme of the review, ‘Lack of support’, focuses on the absence of meaningful support from family and healthcare professionals. This absence is felt during the patient’s illness and after the caregiver has become bereaved. Fourteen of the studies illustrated this theme. This theme incorporates two subthemes: ‘During illness’ and ‘After death’.

#### During illness

This subtheme explores the ‘Lack of support’ experienced by the caregivers while they are providing care to the patient; this exacerbated feelings of loneliness and strained personal relationships. This subtheme was found in 14 studies. A quote from a participant who provided palliative care for her partner^35^ illustrates the subtheme.

That there are not a lot of people who are really there for you. That’s what makes it more difficult, because you really have to do it all alone . . . in the dark and lonely hours, you just can’t phone everybody. (Proot et al.,^35^ p. 116)

Many of the participants were the primary or sole caregivers. They often felt as though their families did not support them enough, leading to feelings of anger and resentment.^32,46^ Some caregivers’ poor family relationships stopped them from sharing their hardships.^28,31,49^ This was particularly difficult for caregivers of patients with HIV. The stigma surrounding the disease caused the family to abandon the patient and caregiver with no support.^31^ For one group of participants, migrant Chinese women living in Australia, the geographical distance from their family and difference in cultural values were isolating.^50^ The lack of support that caregivers received, whether due to physical or emotional distance, intensified during the patients’ dying phase of illness.^51^ Caregivers reported feeling unprepared and overwhelmed by being solely responsible for their loved one dying at home.^48^ Even those with some social support would not ask for help or discuss the impact of caring due to fear of being a burden to others or concerns that other people would not understand their experience.^28,40,46^

The healthcare professionals involved in the patient’s care often did not acknowledge the caregiver.^30,37,40,42,47,52,53^ This made the caregivers feel isolated as they were left out of critical decision-making.^37,49,52^ They could not build relationships with the professionals, which was isolating and reduced the caregivers’ access to important information and guidance.^37,47,52^ This lack of support from healthcare professionals was exacerbated during difficult times such as hospital discharges, where the caregiver felt alone in their responsibilities.^30,53^

#### After death

This subtheme explores the absence of support for the caregiver after the patient has died. This was found in nine studies. Caregivers have insufficient support while grieving and trying to rebuild their lives. Although they are experiencing loneliness as they did during the patient’s illness, the reasons and exacerbating factors have changed. This is demonstrated in a quotation from Linderholm and Friedrichsen’s^37^ study which illustrates both social and emotional loneliness.

No, now I’ve been totally forgotten. After the death of my ex-husband, all the children were altogether here . . . then it when it was all over . . . It feels hard because now I’m not needed anymore. (Linderholm et al.,^37^ p. 33)

Caregivers had fewer people to support them after the patient’s death due to losing social contact over the course of the illness.^36,54^ After the funeral, family and friends provided little support to the caregiver.^41,54,55^ Caregiving had been an all-consuming role. Many caregivers had to cope with the loss of this important role alongside the grief of their loved one.^54,56^ Carers struggled to return to their previous roles and routines. This was especially difficult for caregivers who lost their spouses. They struggled to rebuild their lives, leading to further isolation.^36,40^ Their feelings of loneliness were associated with other negative emotions and health issues, such as sadness, helplessness and hopelessness,^29^ and depression, insomnia and reduced appetite.^37^

Caregivers felt a lack of support from professionals after the patient’s death.^34,55^ They often felt abandoned by people who had significantly impacted their lives. Health professionals provided valuable resources, such as hospice and home-based nursing care.^55^ These palliative care teams were able to provide the carer with support and security during the caregiving period.^34^ However, during bereavement, they lost contact with people who had had an immense impact on their and their loved ones life.^40^ This led to feelings of loneliness and social isolation while grieving.^34,40^

#### What helps?

The third theme of the review, ‘What helps?’, focuses on resources that alleviate feelings of loneliness and social isolation in palliative caregivers. Nine of the studies illustrated this theme. This theme incorporates two subthemes: ‘Support from others’ and ‘Faith and faith communities’.

#### Support from others

This subtheme explores the benefit of support from other people, including family and the patient. This was found in 10 studies and depicted in a quotation from a study of spousal palliative caregivers.^45^

I have two friends; they are like sisters to me. They were there for whatever I needed – financially, as humans, with love, for everything. (Bilić et al.,^45^ p. 11)

Support from family and friends was a valuable resource for caregivers.^35,45,48^ During the patient’s illness, attending external social events and keeping connections with friends and family helped reduce feelings of loneliness.^27,29^ Healthcare professionals’ support helped alleviate feelings of isolation.^35,53^ This was done through task-sharing, being provided with relevant guidance and being included in decision-making.^35,53^ The patients were also a source of support for the caregivers.^45,53^ Caregivers reported that the inevitability of the patient’s death and increased time together deepened their relationship.^53,56^ Caregivers and patients wanted to make the most of their time together.^48^ This is greatly contrasted with experiences of social isolation and loneliness.

After the patient’s death, family and friends helped reintegrate the caregivers into their wider community.^48^ However, for some, they did not have the social support they hoped for, leading them to feel disappointed and lonely.^31^

#### Faith and faith communities

The final subtheme illustrates the importance of faith and faith communities for some caregivers in their journey of providing palliative care. It was found in four studies. This is illustrated in a quotation from a daughter who provided end-of-life care.^41^

My faith increased . . . God took something from me, but he gave more back . . . He showed me every day that it’s not just me who goes through this. (Benites et al.,^41^ p. 252)

For some caregivers, their faith helped them cope with the approaching death and then the loss of their loved one.^41^ Their devotion to the patient was aided by their religious convictions.^41,53^ Faith communities were a vital support system.^29,31^ Prayers provide connection and relationship-building to reduce loneliness.^31^ In contrast, a few caregivers reduced their faith practices due to their disappointment, as they felt their prayers had not been answered.^41^

## Discussion

This systematic review aimed to explore the experience of loneliness and social isolation within the occupational role of being an informal palliative caregiver. The findings of this review indicate that providing informal palliative care can lead to many experiences that develop or heighten feelings of loneliness and social isolation. Firstly, one of the main findings was the complexity of palliative caregiving. Caregiving is prioritised over the needs of the caregiver. Palliative caregivers often do not share their burdens or the seriousness of the patient’s condition. They lose the patient’s companionship, particularly as their symptoms worsen. Secondly, the review found that a lack of support during the patient’s illness led to the caregiver experiencing loneliness and social isolation. The toll of palliative caregiving extends into bereavement as shown by the loneliness and social isolation experienced by caregivers after the patient’s death. This was due to many interconnected factors, such as poor service provision, strained familial relationships and geographical distance. Finally, the review found resources that help to alleviate loneliness and social isolation. This included support from the patient, family, friends and healthcare professionals. For some caregivers, their connection to their faith and faith communities relieved loneliness and increased their devotion to caring for the patient.

The findings of this systematic review align with and build upon previous theoretical frameworks, including those of caregiver burden and caregiver burnout. The concept of caregiver burden is defined as the multidimensional strain perceived by caregivers when providing care for a loved one.^57^ In a concept analysis, loneliness and social isolation were cited as antecedents to caregiver burden, due to disruptions in their lifestyle.^57^ This closely links to the findings of the current that the priority of caregiving over one’s own social needs is a common experience among palliative caregivers. Similarly, in a theoretical framework of caregiving burnout, reduced social life was proposed as a major stressor, which require support groups and increased regular social contact to reduce caregiver burnout.^58^ These experiences are evidenced in findings across the current review’s themes.

Importantly, this review builds on the findings of previous palliative care research. Dahlborg Lyckhage and Lindahl^9^ studied six palliative caregivers, focusing on liminality and its effect on their self-image. They found that the participants did not feel they could put themselves first, leading to loneliness.^9^ They felt guilty if they experienced any joy.^9^ This is similar to the results of this review, which found that as caregiving is prioritised, the caregivers lose social contacts and feel lonely. A further similarity was that the caregivers felt abandoned with too much responsibility to provide palliative care.^9^ This was found in this review, as caregivers reported a lack of support from family and healthcare professionals during the patient’s illness. In Reigada et al.,^18^ systematic review of the role of the palliative caregiver, they found that part of the role includes being constantly available to the patient, keeping the patient’s hopes up and supporting the remaining family members. These findings align with the results of this review, which found that caregiving is prioritised over the caregivers’ needs and that the caregiver takes responsibility for protecting the others emotionally. The current review also adds to the understanding of the unique experiences of palliative caregivers. As found in Reigada et al.^18^ review, there are complex and difficult situations associated palliative caregiving that impact the caregiver. These specific tasks and requirements of an informal palliative caregiver can lead to experiences of loneliness and social isolation that are particular to this type of care.

The findings of this review also extend to previous studies that have explored loneliness in informal caregiving. In a narrative review of loneliness in cancer caregivers, Gray et al.^20^ found that caregiving limits discretionary social contacts and exhaustion from caregiving, leading to feelings of loneliness. This was similarly explored in this review, which found that caregiving is prioritised over socialising, and the burden of caregiving alone is overwhelming. Chistell et al.^59^ found that the participants experienced loneliness due to abandoning previous occupations and limited energy. They also found that as the patient’s dependency on the caregiver increased over time, this altered their relationship and led to loneliness. This is in line with the results of this review; as the patient’s symptoms worsen, their dynamic changes and previous roles, such as parent and child, are lost. In contrast to this, they found that for some caregivers, their relationship with the patient intensified, and their loneliness was alleviated through connection.^59^ This experience was captured in the findings of this review; for some caregivers, the patient was a vital social support.

With regards to practices that help to alleviate loneliness, the review’s findings align with previous research. In a systems approach to social connection, Holt-Lunstad^60^ argues that social integration and support are protective factors against the health risks associated with loneliness and social isolation, which is referred to as the stress buffering effect of social support. Similarly, in an integrated review of burden in palliative care,^61^ argue that perceived social support can protect from the pathogenic effects of burden. This review found that some palliative caregivers found social connections and faith communities reduced feelings of loneliness and social isolation. This included maintaining previous hobbies and social events, attending bereavement support groups, and group prayer with those of the same faith. Similarly, Gray et al.’s^20^ narrative literature review concluded that befriending programmes can reduce loneliness, improve emotional wellbeing and re-engage cancer caregivers in their communities. They also found that group-based activities can increase a sense of belonging and promote social support.^20^ Thus, engagement with befriending services and group programmes may be useful practices for palliative caregivers to alleviate loneliness and social isolation. However, barriers to such interventions include the caregiver’s lack of time and limited respite provision.^20^ This is also reflected in this review, which found that a lack of support from healthcare professionals and poor service provision were common in the experience of loneliness and social isolation.

The practices of healthcare professionals can help alleviate loneliness and social isolation in palliative caregivers. The results of this review suggest that healthcare professionals can reduce loneliness and social isolation by task-sharing and including caregivers in decision-making. This is in line with Chistell et al.’s^59^ study, which suggests that healthcare professionals should recognise and value the expertise of caregivers. Developing a supportive relationship is essential in countering loneliness in caregivers.^59^ Similarly, in the systematic view conducted by Reigada et al.,^18^ they concluded that ensuring caregivers receive information in a timely manner can promote the feeling of inclusion in the healthcare team. Thus, it may be useful for health professionals to meaningfully involve the caregivers in patient care by providing timely and accessible information, supporting them with practical tasks and recognising their expertise. In these ways, health professionals may be able to alleviate some loneliness and social isolation experienced by palliative caregivers. Although there are similarities to previous empirical research and reviews, this review is the first, to our knowledge, to specifically explore loneliness and social isolation in the context of palliative caregiving.

### Limitations

Due to limited resources, the review only included studies that had been published in English. Of the 28 studies reviewed, 86% were from Western countries, despite the researcher’s aim to report on global experiences of loneliness and social isolation in palliative caregiving. Therefore, it is possible that valuable findings were absent. However, the researcher used a comprehensive research strategy to ensure that a large range of studies were included.

It should be noted that loneliness and social isolation were defined and operationalised differently across the studies included in this review. This did limit the ability of the current study to analyse and explore the concepts separately. However, the inclusion of both concepts in the current study demonstrates their interconnectedness.

A strength of the review was the use of reflexivity throughout the reviewing process. Reflexivity was used in discussions with the second author about the first author’s personal experience of being a palliative caregiver. This allowed continuous self-reflection and awareness of the first author’s subjective role in areas such as designing the review and analysing the data.^62^

### Implications of findings

The findings of the review have implications for future practice in palliative care. For example, professionals could work with the caregivers before and after the patient’s death to help them develop meaningful occupations that reintegrate them into their community and allow for social connection. This may specifically address social isolation for palliative caregivers. Another example is for professionals to encourage and aid, when relevant to the carer, engagement with their faith/religious practices and communities as a resource for emotional strength and support. This may be able to address feelings of loneliness experienced by palliative caregivers. A systematic lack of spiritual care has been identified as an issue, by healthcare professionals working with palliative carers.^63^ Thus, a wider cultural shift towards acknowledging and supporting individual’s religious and/or spiritual practices could benefit many carers experiencing loneliness and social isolation.

Future research should aim to investigate effective interventions that alleviate loneliness and social isolation in palliative caregivers. It may be beneficial for future research to use a longitudinal design to explore when interventions are most valuable during the journey of providing palliative care. Longitudinal research could also be used to investigate how the reasons for loneliness and social isolation change across different phases of palliative caregiving. This is because the reasons for caregiver loneliness and social isolation change at different stages of palliative caregiving; therefore, interventions may need to be altered to reflect this. Any future research into this topic may benefit from separating the constructs of loneliness and social isolation more distinctly. This could be done through use of measures such as the De Jong Gierveld Loneliness Scale, which measures social and emotional loneliness, and the Lubben Social Network Scale, which measures size, frequency and closeness of social contacts.^4^

## Conclusion

The findings of this review illustrate the complexity of providing informal palliative care and how this can lead to caregivers experiencing loneliness and social isolation before and after the patient’s death. The implications of the study include recommendations for practitioners working with palliative caregivers and future research that assesses interventions to alleviate loneliness and social isolation in palliative caregivers.

## Supplemental Material

sj-docx-1-pcr-10.1177_26323524251397000 – Supplemental material for Exploring the lived experience of loneliness and social isolation in informal palliative caregivers: A systematic review Supplemental material, sj-docx-1-pcr-10.1177_26323524251397000 for Exploring the lived experience of loneliness and social isolation in informal palliative caregivers: A systematic review by Louisa Cole, Tracy Collins, Renée Speyer, Cara Ellis and Reinie Cordier in Palliative Care and Social Practice
